# School and Home

**Published:** 1905-01-28

**Authors:** 


The Hospital.

A Journal of

The Medical Sciences and Hospital Administration.

Vol. XXXVII.—No. 957. JANUARY 28, 1905.

### School and Home.

Many years ago, before the education of the children of the labouring classes was either enforced by law or paid for by the community, one of her Majesty Queen Victoria's Inspectors of Schools propounded, in his annual report, what a citizen of the United States might possibly have described as a "conundrum." "Of what use," he asked, "is education to a child, if the interval of a single school vacation is to be accepted as a sufficient excuse for his having totally forgotten the instruction he has received?" We are beginning, in the present day, to entertain modified views of the proper subjects and proper methods of instruction, and to perceive the imperative necessity of bringing into the curriculum a certain amount of teaching of hygiene and of sanitation, with the one consequence, among others, that we are confronted by the inspector's query in a somewhat different form. Of what use is the teaching, if the conditions existing in the homes of the learners are of such a kind as to render its application impossible? An old saying, usually perverted in common use, tells us that "cleanliness is next to goodliness," or, in other words, that, if people cannot be "goodly," *i.e.* beautiful, at their pleasure, they may at least be clean; and we know, of course, that cleanliness is one of the most important factors of health in crowded communities. It will be an important duty of the hygienic teacher to impress this principle upon his pupils, and to render a belief in the virtues of soap and water one of the chief articles of their faith. Unless the teaching is to be absolutely thrown away, the community which insists upon it must insist also, and with equal pertinacity, upon the maintenance of domestic conditions which render the use of soap and water practicable. These conditions, in their simplest form, are that clean water should be rendered readily accessible in every home, and that convenient outlets for soiled water should be provided. The late Dr. Vivian Poore once drew a graphic picture, as a result of his inspection of some tenement houses not far from Oxford Street, of the difficulties which interposed themselves between the wives of the tenants and the fulfilment of any lingering desires after cleanliness which circumstances had permitted them to retain. He took the case of a working man's wife whose tenement was on the fifth or sixth floor of a lofty house. Her only source of water supply was from a stand-pipe in the back yard. At the time of Dr. Poore's visit she had three children and was six months advanced in pregnancy. Her only vessel for carrying water from the stand-pipe to her domicile was a pitcher the handle of which had been broken off, and which was furnished with a substitute made of string. Her only vessel for storage, except the pitcher itself, was a shallow earthenware pan with no cover, in which the water was exposed to various kinds of contamination, atmospheric and accidental, and from which it could only be obtained by baling, so that a little dirt was always left at the bottom to pollute the next supply. Soiled water and domestic slops had to be carried down stairs with the same labour that was required for bringing fresh water to the top. The woman had been a domestic servant, accustomed as such, to at least some semblance of personal and household cleanliness, but, as a matter of necessity, she had degenerated into a filthy slattern, and her house and her children were as dirty as herself.

The leading features of Dr. Poore's graphic description have been vividly recalled to us by a perusal of a report from Dr. Young, on tenement houses in the metropolis, which forms the second Appendix to Sir Shirley F. Murphy's Report for 1903 to the Public Health Committee of the London County Council. There are in London a very large number of streets in which the houses were originally constructed with a view to occupation by single families, but in which they have fallen from this estate, and are now let out in tenements, or even in single rooms. This condition seems to be brought about in two different methods, sometimes by the tenant of the whole house sub-letting, and sometimes by direct contract between the tenement-holders and the landlord. A house let in tenements, when certified by the local medical officer of health as fitted for this purpose, is exempted from the inhabited house duty; and this exemption is largely sought and obtained, although it seems very probable that the necessary certificate is often granted upon insufficient grounds. The only statutory requirement seems to be such a construction of additional closet accommodation as may afford at least one closet for every 12 occupants; and neither the supply of water to each floor nor the provision of proper waste pipes is insisted upon. The Metropolitan Branch of the Society of Medical Officers of Health has laid down certain requirements which, in the opinion of the society, should be fulfilled before certificates conferring exemption from house duty are given; but, in the case of many of the houses to be dealt with, these requirements could not be fulfilled except by entire reconstruction, and hence, in many localities, they are not insisted upon. The general result, of course, except that the houses, as a rule, are not more than four storeys high, is a practical reproduction of the condition which Dr. Poore described; a condition in which neither personal nor domestic cleanliness is possible, and in which, therefore, it soon ceases to be striven for. The sanitary teaching of the school, on which so much of the physical condition of the coming generations is to depend, is in danger of being rendered wholly nugatory by the conditions of home; and the self-respect which is one of the effects of cleanliness will be effectually prevented from coming into existence. It is obvious that even a good domestic supply of cold water would be only a partial remedy for the evils which now exist, and that facilities for obtaining hot water at a cheaper rate and in greater abundance than from tea kettles are essential to the cultivation of anything like cleanly habits among the working classes of the metropolis. It is far easier to point out defects than to suggest remedies at once adequate and practicable; but the conditions described by Dr. Young constitute a very grave evil, and one certain to exert a definitely prejudicial influence upon large classes of the population. Probably a greatly increased stringency of the law, combined with a somewhat wide discretion to local authorities in applying it, might lead the way to a better condition of things hereafter.

